# Gravity Reduced Nitrogen Uptake *via* the Regulation of Brace Unilateral Root Growth in Maize Intercropping

**DOI:** 10.3389/fpls.2021.724909

**Published:** 2021-09-06

**Authors:** Guopeng Chen, Bing Liang, George Bawa, Hong Chen, Kai Shi, Yun Hu, Ping Chen, Yuanfang Fan, Tian Pu, Xin Sun, Taiwen Yong, Weiguo Liu, Jiang Liu, Junbo Du, Feng Yang, Xiaochun Wang, Wenyu Yang

**Affiliations:** ^1^College of Agronomy, Sichuan Agricultural University, Chengdu, China; ^2^Sichuan Engineering Research Center for Crop Strip Intercropping System, Key Laboratory of Crop Ecophysiology and Farming System in Southwest China, Ministry of Agriculture, Chengdu, China

**Keywords:** intercropping, plant inclination, indole-3-acetic acid, root morphology, nitrogen uptake

## Abstract

Water, nutrient, light, and interspecific facilitation regulation of soil physicochemical properties and root morphology modulate nitrogen (N) uptake in cereal and legume intercropping systems. However, maize root morphological plasticity and N uptake capability response to gravity in the intercropping system remains to be determined. In this study, maize was grown under 20 cm (I_20_), 40 cm (I_40_), and 60 cm (I_60_) of narrow row spacing in an intercropping system (maize–soybean strip relay intercropping) and equal row spacing of monoculture (M) in a 2-year field experiment. As a supplementary for the field experiment, maize root barrier and plant inclination experiments were conducted. Plant inclination, brace root morphology, N uptake, indole-3-acetic acid (IAA) level, IAA synthesis genes, and grain yield were assessed. The result showed that the plant inclination increased with decreasing narrow row spacing in intercropping system. Also, the brace unilateral root growth ratio (BURR) increased with increasing plant inclination in intercropping treatments. The plant inclination experiment showed the BURR achieved 94% after inclination at 45°. BURR tended to be positively correlated (*p* = 0.00) with plant inclination. Thus, gravity (plant inclination) causes brace unilateral root growth. The IAA concentration of stem nodes in the wide row increased with increasing plant inclination, while the IAA accumulation decreased in the narrow row. The *Zmvt2* and *ZM2G141383* genes (associated with IAA biosynthesis) were highly expressed in a wide row. There was a strong correlation (*p* = 0.03) between the IAA concentration of wide row and the BURR. Therefore, gravity regulates the IAA level, which affects BURR. In addition, the brace root number, volume, and surface area were decreased when BURR was increased. Subsequently, the leaf N, cob N, and kernel N accumulation were reduced. These organs N and grain yield in I_60_ were not significantly different as compared to the control treatment. The excessive brace unilateral root growth was not conducive to N uptake and increased yield. Our results suggest that gravity is essential in regulating root morphology plasticity by regulating IAA levels and decreasing N uptake capacity. Furthermore, these results indicate that plant inclination can regulate root phenotype and N uptake of maize and by adjusting the spacing of narrow maize row, we can improve the N uptake and yield of the maize–soybean strip relay-intercropping system.

## Introduction

It is assumed that the world population might reach 9.3 billion by the year 2050, which indicates that an increase in food production with limited resources is urgent for the growing population (Xie et al., 2017). In the last two decades, intercropping approaches have increased crop yields, land-use efficiency, light and soil nutrients (Long et al., 2001; Kermah et al., 2017; Wang et al., 2017). The mechanism underlying how nitrogen (N) acquisition increases intercropping species, particularly cereal–legume systems, is demonstrated by nitrogen-fixing legume and belowground interspecific facilitation. Intercropping maize enhances nitrogen fixation of faba bean by maize root exudates, enabling flavonoid synthesis in faba bean that increases nodulation (Li et al., 2016). Furthermore, cereals can stimulate nodulation and N_2_ fixation by legumes, presumably through competition for nitrate or ammonium in the rhizosphere (Jensen, 1996; Neumann et al., 2007). Moreover, intercropping system enhances N uptake by highly plastic root morphology and fungal diversity through interspecific interactions between cereal and legume (Ramirez-Garcia et al., 2015; Zhang et al., 2020). However, except for these findings, detailed investigations on how the environment (excluding the interspecific interactions) regulates root developmental plasticity to increase cereal N uptake is lacking. Although light, water, and nutrient spatial distribution in intercropping are different from monoculture (Gao et al., 2010b; Liu et al., 2017; Rahman et al., 2017; Zhou et al., 2019a).

Light, root gravitropism, and chemotropism play a vital role in the direction in which roots elongate. The primary effect of light is enhanced photosynthesis, which leads to sucrose production that enables root growth (Kircher and Schopfer, 2012). Under low-light intensity, photosynthates were distributed to shoots to enhance the interception of light, and that the ratio of root to shoot biomass decreased (Gommers et al., 2013; Gundel et al., 2014). In addition, photomorphogenic development is associated with the production of auxins in the young shoot tissues, which are transported to the root, thereby enabling root development (Reed et al., 1998; Bhalerao et al., 2002). In the interaction of light and gravitropism, light-induced changes in indole-3-acetic acid (IAA) levels and distributions negatively regulate maize root phototropism and gravitropism (Burbach et al., 2012; Suzuki et al., 2016). The changes in IAA level due to light and gravitropism interaction could be due to the changes in expression levels of auxin biosynthesis genes (Du et al., 2013). Root in water has an unequal potential condition and cytokinins are increased at the lower water potential side, which upregulates the expression of *ARR16* and *ARR17*, leading to increased cell division at the lower water potential side of the root tip. As a result, the root tip bends toward the higher water potential side of the root (Chang et al., 2019). Furthermore, root systems acquire nitrogen through the uptake of nutrients such as nitrate from the soil. Ammonium, nitrate–nitrogen can be distributed heterogeneously in the soil. Local N deficiency prevents lateral root growth and activates peptides production of C-terminally encoded peptide (CEP); these are perceived in the shoot by LRR–RK receptors, leading to an unknown systemic signal that can stimulate growth in the soil high-N areas (Bisseling and Scheres, 2014). Therefore, environmental factors influencing root growth or root morphology affect nutrient uptake (Li et al., 2011; Cheng et al., 2014).

Maize–soybean strip relay-intercropping (MSR) is an efficient and sustainable cropping system with high land equivalent ratio, N, and phosphorus use efficiency that has been widely expanded in China (Feng et al., 2014; Liu et al., 2017; Raza et al., 2019). MSR utilized wide-narrow row planting and fertilizer application in a wide row that resulted in light, water (rainfed), and nutrient heterogeneously distribution in both the sides of maize plants (Liu et al., 2017; Rahman et al., 2017; Yong et al., 2018; Chen et al., 2019, 2020). In MSR, different works showed that the plant root grows toward the wide row (the row between cereal and legume) due to the dominance of the microenvironment and interspecific interactions in the wide row (Gao et al., 2010a; Yang et al., 2010; Ren et al., 2017). However, plants readjust their growth direction by sensing any deflection relative to the direction of gravity vector (gravitropism), which enables the shoot in a best positioned to utilize its light interception capabilities to allow further uptake of water and nutrients (Singh et al., 2014; Nakamura et al., 2019). Thus, plants in high density grow toward one side of the interrow space, while neighboring plants grow in opposite directions. These responses are regulated by the alterations in the red/far-red ratio of the light perceived by phytochrome interacting factors (PIFs) (Lopez Pereira et al., 2017). Here, a similar phenomenon was observed; the plants inclined toward the wide row in intercropping maize. Therefore, we hypothesized that when maize plant deflects toward the wide row, IAA accumulates in a wide row, and brace root responds to the direction of gravity by modulation of root system growth patterns and plasticity, which affects plant N uptake. Thus, a long-term field experiment with MSR and two control environment experiments were conducted to investigate how gravity stimulates root morphology and physiology and the effects of plasticity on N uptake. Again, this study investigated whether gravity regulates brace root morphology by IAA unequal distribution and the impact of uptake capacity of N in intercropping system.

## Materials and Methods

### Experimental Design

Study 1 (intercropping and monoculture root comparison experiment). A 2-year field experiment was conducted in the maize growing season from 2019 to 2020, in which two cultivation patterns were compared. A randomized complete block design with three replications was used in this experiment due to drainage differences in a field under rainfed conditions. The maize–soybean relay intercropping was narrow–wide row (40 + 180 cm) pattern (I_40_), maize and soybean row ratio 2:2, a monoculture of maize with equal row spacing (70 + 70 cm) as a control (M) at the Renshou experimental site (2019) in Sichuan Province, China (29°60′N, 104°00′E). The characteristics of the soil of the experimental site have been reported (Zhou et al., 2019c). Two treatments were added in the 2020 experiment, the narrow and wide row spacing of maize was 20 + 200 cm (I_20_), 40 + 180 cm (I_40_), and 60 + 160 cm (I_60_) in maize–soybean intercropping, a monoculture of maize was used as a control (M) (Supplementary Figure 1). The density of six plants m^–2^ of maize (*Zea mays* L. cv. Zhongyu 3), intercropping, and monoculture plant spacing were 15 and 24 cm, respectively, and each treatment had three replicates (three plots), and each plot was 40 m^2^ (6 m × 6.6 m), in total, there were 12 plots. Three plants were used in each plot, and nine plants were used in each treatment. Maize seed was sown on March 28, 2019 and April 9, 2020, and harvested on August 1, 2019, August 2, 2020, respectively. The maize fertilizer consisted of 600 kg ha^–1^ of superphosphate (P_2_O_5_ 12%), 150 kg ha^–1^ of potassium chloride (K_2_O 60%), and 180 kg ha^–1^ of nitrogen (N) in the whole growth period of the maize. The base fertilizer:topdressing (10 days before flowering) was 5:5. The fertilizer was applied to the maize wide row (a distance of 15 cm between the maize plant and the fertilizer). Soybean (*Glycine max* L. cv. Nandou 12) seed was sown on June 16, 2019 and June 19, 2020 with a density of 12 plants m^–2^.

Study 2 (maize root barrier experiment). The root barrier experiment was performed to limit the effects of interspecific interactions and root chemotaxis, ensuring nutrient and relative distribution of water around the root system. The wooden frames (bottomless, length 35 cm × width 15 cm × height 35 cm) were buried in the soil, and then the seeds were sowed in the wooden frames. A randomized complete block design with three replications was used because of the drainage differences in a field under rainfed conditions. The planting pattern, density, variety, and sowing date were the same as in Study 1 in 2020. Treatments were wide–narrow row spacing of maize 20 + 200 cm (SI_20_), 40 + 180 cm (SI_40_), 60 + 160 cm (SI_60_) in maize–soybean intercropping, respectively, with monoculture of maize as a control (SM) in Supplementary Figure 2. The 10 g superphosphate (with P_2_O_5_ 12%), 2.5 g potassium chloride (with K_2_O 60%), and 18.9 g urea (with N 46%) were applied to each plant with fertilizer spread evenly on the frames. The base fertilizer:topdressing (10 days before flowering) was 5:5. The soybean sowing date and density were the same as that of Study 1 in 2020.

Study 3 (plant inclination experiment). To further verify the effects of plant inclination on brace root growth, we designed this trial. A completely randomized experimental design was used. Monoculture maize, row spacing 200 cm, plant spacing 15 cm, and fertilizer were applied around the plant (Supplementary Figure 3). The plot was 24 m^2^ (4 m × 6 m), 80 plants. Six plants were randomly selected as one group (total of three groups), pulled with a rope, and tilted to 45° (L_45_) at stage V14 (when the uppermost whorls of the brace root began to grow) in every row with plants growing upright as control (L_0_). The sowing date and variety were the same as in Study 2.

### Measurement of Plant Inclination and BURR

Plant inclination in Studies 1 and 2 was performed with an electronic protractor (500, BRT, China). The plumb was perpendicular to the ground. We measured the angle between the stem and the line of plumb at stage V14. In each plot, the brace unilateral root growth ratio (BURR) and brace root number of narrow and wide rows (or left and right of monoculture) in all the studies were calculated at 1 day after flowering (1 DAF). In Studies 1 and 2, the calculation formula was BURR = the number of plants with brace unilateral root growth / the number of plants in each plot. Each plot had 240 plants in Studies 1 and 2. In Study 3, the calculation formula was BURR = the number of plants with brace unilateral root growth / 6.

### Root Morphology Examination

Brace roots in Study 1 were recovered from the soil and washed thoroughly with water at 25 days after flowering (25 DAF). Three representative plants (not taken from the plot edge, disease-free, and healthy plant) from each plot were sampled. The narrow and wide rows brace roots and the whole root was harvested separately by using a borehole. The brace root was dipped in water and positioned on a square dish to reduce root overlapping and then captured using an Epson V700 scanner (Japan). Root volume and surface area were analyzed with WinRHIZO (WinRHIZO Pro2004, Canada) software (Zhou et al., 2019b). The biomass of brace and total roots (including primary root, brace roots, and lateral roots) were separately collected after the scanning and dried to constant weight in an oven at 85°C for 1 week.

### Organs Nitrogen Level, Dry Weight, and Yield Assays

Nitrogen content assay was determined by using an elemental analyzer (rapid N exceed, Elementar, Germany). Dry weight was measured from three randomly selected plants in each plot at 1 DAF and maturity in Study 1. Leaf, stem, cob, and kernel were oven-dried at 85°C to a constant weight. Organs were pulverized, 120 mg of the samples were transferred into tin capsules, and N ratios were analyzed. At maturity, the formula for N accumulation, harvest index (HI), and yield was as follows: organ N accumulation = organ N ratio × organ weight, HI = kernel weight / dry weight, two rows of plants were selected in each plot for the calculation of effective ears and yield, grain yield = effective ears × grain number per ear × 1,000-grain weight (Raza et al., 2019).

### Indole-3-Acetic Acid Concentration and Key Biosynthesis Pathway Genes Analysis

In each plot, three plants were selected at the V14 stage when the plants began to incline (Supplementary Figure 5). When the first brace root layer of the aboveground started to grow, stem nodes samples from left and right of the stem with the first node of the aboveground (stem node differentiation brace root) were collected and used for hormone extraction and gene expression analysis in Study 1. High performance liquid chromatography-mass spectrometry/mass spectrometry (HPLC-MS/MS) was used to determine the IAA content. The standard IAA was purchased from Sigma-Aldrich (MO, United States). Briefly, 0.5 g of sample was weighed and a 10-fold volume of acetonitrile was mixed in a centrifuge tube. The mixtures were centrifuged at 12,000 *g* for 5 min at 4°C and the supernatants were collected. Next, the sample was precipitated by fivefold volume. The extraction was repeated two times and the supernatant was collected. Finally, the sample was dried using nitrogen gas and redissolve with 200 μL methanol and filtered with a 0.22 μm organic filter membrane for HPLC–MS/MS analysis (Splivallo et al., 2009).

Total RNA was extracted using an RNA isolation kit (RNeasy Plant Mini Kit, QIAGEN), and cDNA was synthesized and quantitated by real-time reverse transcription-PCR (Thermo Fisher Scientific, MA, United States). Quantitative PCR (qPCR) was performed in Quant Studio 6 Flex real-time PCR system (Thermo Fisher Scientific, MA, United States). The parameters of the semi-quantitative PCR were as follows: 95°C for 5 min, 95°C for 15 s, 50°C for 30 s, 72°C for 1 kb min^–1^, moved to step 2 for another more cycles according to the level of the specific genes. Parameters of the qPCR were as follows: 95°C for 3 min, 95°C for 15 s, 55°C for 15 s 72°C for 20 s, moved to step 2 for 39 more cycles. The increment of 0.5°C from 65 to 95°C for 5 s was used for melt curve analysis. The data analysis was conducted using the 2^–ΔΔCT^ method. The determination of Cycle threshold (C_t_) values was performed by subtracting the levels difference of Ct (Zhou et al., 2019b; Bawa et al., 2020, 2021). The *ZmACTIN*–F-(TCACTACGACTGCCGAGCGAG)-R-(GAGCC ACCACTGAGGACAACATTAC) gene was used as a housekeeping gene. The trp aminotransferase-related gene (*Zmvt2*) and YUCCA genes (*ZM2G141383* and *ZM2G019515*) were investigated. The primer sequences are described in Supplementary Table 1.

### Statistical Analysis

Analysis of variance was used to determine significant differences in Studies 1, 2, and 3. The variance was analyzed for each year in Study 1 independently. Least significant difference (LSD) test was used *post hoc* testing of differences between treatments (2019, Origin Lab Corporation, Northampton, MA, United States). A *p* < 0.05 was considered to be significant in three studies. Pearson’s correlation test was used to analyze the correlations.

## Results

### Plant Inclination, Root Morphology, N Accumulation, and Yield in 2019

**FIGURE 1:**
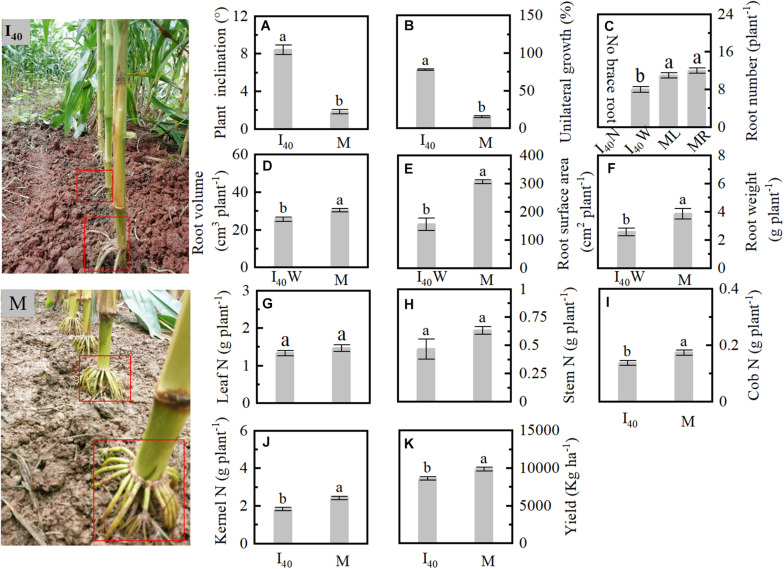
Plant inclination, root morphology, N accumulation of organs, and yield in Study 1 in 2019. **(A)** Plant inclination at stage V14 (14th leaf stage). **(B)** Brace unilateral root growth ratio. **(C–F)** Brace root number, volume, surface area, and weight per plant at 25 days after flowering (25 DAF). **(G–J)** The N accumulation of leaf, stem, cob, and kernel per plant at maturity. **(K)** Kernel yield at maturity. I_40_: the narrow (N) and wide (W) row spacing of 40 + 180 cm in intercropping. ML and MR: the left (ML) and right (MR) sides of the maize plant of monoculture (M). Data with the different letters are significantly different (*p* < 0.05).

We analyzed the intercropped and monoculture maize morphology and physiological parameters in Study 1 in 2019 (Figure 1). Plant inclination and BURR in I_40_ were 367, 409% higher than in M, respectively (Figures 1A,B). The brace root number, volume, and surface area of wide row were significantly lower than in the M left (ML) and right (MR) (Figures 1C–E). Cob and kernel N in I_40_ were reduced than in M (Figures 1I,J). Finally, the grain yield was lower than in the M (Figure 1K).

### Plant Inclination

**FIGURE 2:**
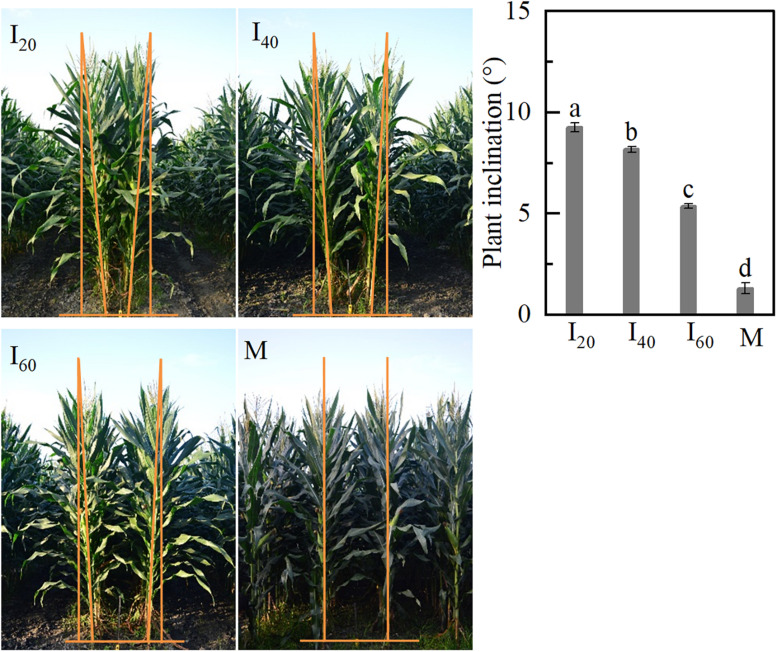
Plant inclination in Study 1 (2020). I_20_, I_40_, and I_60_ represent the intercropping maize planted in narrow–wide row spacing patterns, narrow and wide row spacing of 20 + 200, 40 + 180, and 60 + 160 cm, respectively. M: monoculture maize with a row spacing of 70 cm. Data with different letters are significantly different (*p* < 0.05).

Although we found that brace unilateral root growth and N accumulation decreased in intercropping (narrow-row spacing 40 cm) maize at plant inclination, the evidence was imperfect in 2019. Therefore, two treatments (narrow-row spacing 20 and 60 cm) were added in intercropping maize for auxin detection in Study 1 in 2020. For intercropping maize, plant inclination increased with decreasing narrow-row spacing (Figure 2). I_20_ was higher by 12 and 41%, compared with I_40_ and I_60_ groups. The plant inclination of M (1.3°) was significantly lower than I_20_ (9.3°). The intercropping plants tilted toward the wide row inclination.

### BURR and Morphology

**FIGURE 3:**
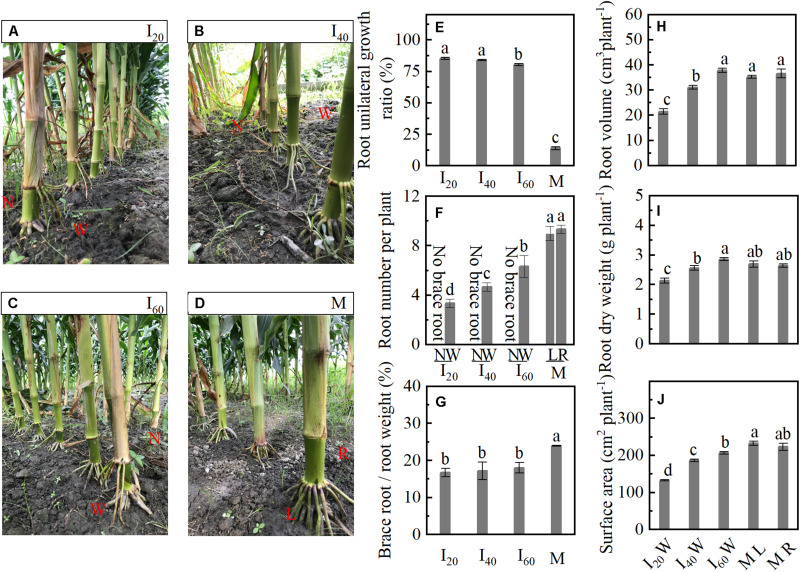
Unilateral root growth ratio and morphology in Study 1 (2020). I_20_, I_40_, and I_60_ represent the intercropping maize planted in narrow–wide row spacing patterns, narrow and wide row spacing of 20 + 200, 40 + 180, and 60 + 160 cm, respectively. M: monoculture maize with a row spacing of 70 cm. ML: the left side of monoculture plant; MR: the right side of monoculture plant. **(A–D)** The narrow row (N) and wide row (W) brace root of I_20_, I_40_, I_60_, and monoculture brace root character, respectively. **(E)** Brace root unilateral growth ratio; **(F)** the number of narrow-row and wide-row brace root; **(G)** the ratio between the brace and total root (including primary root, brace roots, and lateral roots). Panels **(H–J)** represent volume, dry weight, and surface area of brace root in wide row per plant, respectively, narrow-row no brace root, only show data for wide-row brace root. At 25 days after flowering. Data with different letters are significantly different (*p* < 0.05).

The BURR of I_20_ was 8 and 86% higher than I_60_ and M (Figures 3A–E). Compared with ML and MR, the number of brace roots was significantly decreased in I_20_ wide row (I_20_W), I_40_ wide row (I_40_W), and I_60_ wide row (I_60_W). We did not observe any significant difference between ML and MR (Figure 3F). In addition, with the decrease in narrow-row spacing, the root volume, dry weight, and surface area also decreased gradually (Figures 3H–J) at 25 days after flowering (25 DAF) with no significant difference between ML and MR, but was increased in M than intercropping maize (I_20_W, I_40_W, and I_60_W). The ratio between the brace and total root weight of I_20_, I_40_, and I_60_ was more than 17%, M reached 24%, I_20_, I_40_, and I_60_ with a similar ratio lower than M (Figure 3G). Thus, the brace unilateral root growth (only growth in wide row) under intercropping maize and brace root decreased.

### Nitrogen Accumulation in Respective Organs

**FIGURE 4:**
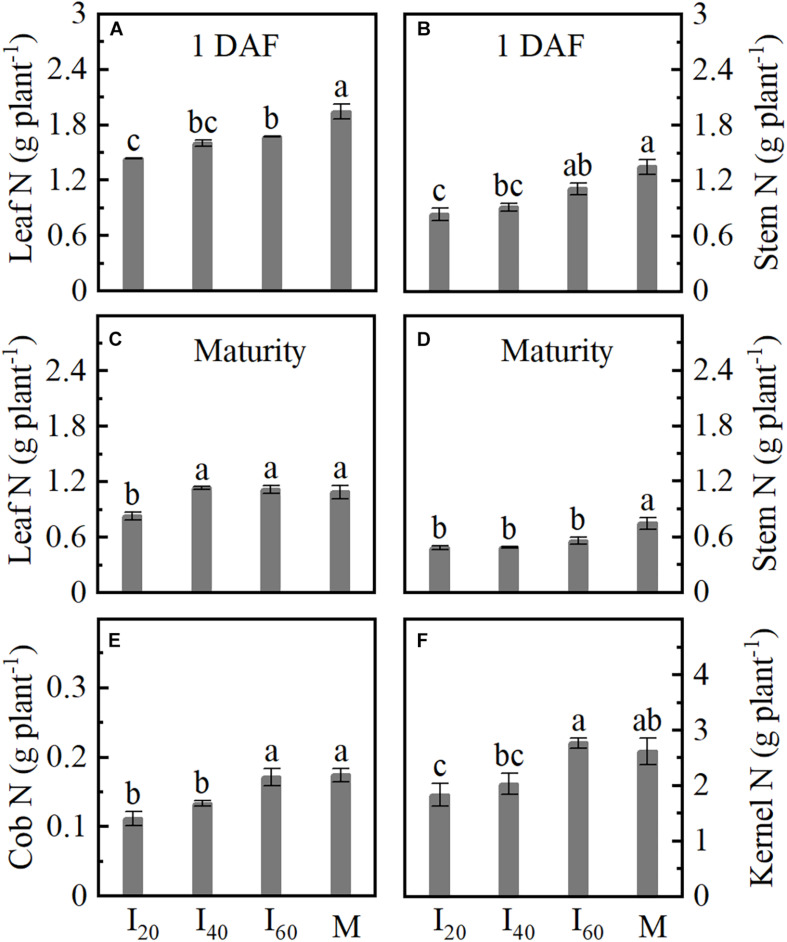
Nitrogen (N) accumulation in respective organs in Study 1 (2020). I_20_, I_40_, and I_60_ represent the intercropping maize planted in the narrow–wide row spacing patterns, narrow and wide row spacing of 20 + 200, 40 + 180, and 60 + 160 cm, respectively. M: monoculture maize with a row spacing of 70 cm. **(A)** Leaf N uptake at 1 day after flowering. **(B)** Stem N uptake at 1 day after flowering. **(C–F)** The leaf, stem, cob, and kernel uptake at maturity. Data with the different letters are significantly different (*p* < 0.05).

Leaf, stem, cob, and kernel nitrogen (N) uptake were reduced in intercropping maize compared with monoculture. In Figures 4A,B, leaf and stem N in I_20_ at 1 day after flowering (1 DAF) were significantly decreased by 26 and 38%, respectively, compared with the I_60_. In addition to the N decrease of leaf and stem, there were reduced cob and kernel in I_20_ and I_40_ at maturity (Figures 4C–F). The cob and kernel N were not significantly different between I_60_ and M. Overall, we observed that N uptake decreases with reducing narrow-row spacing in the intercropping.

### Dry Weight, HI, and Kernel Yield

**FIGURE 5:**
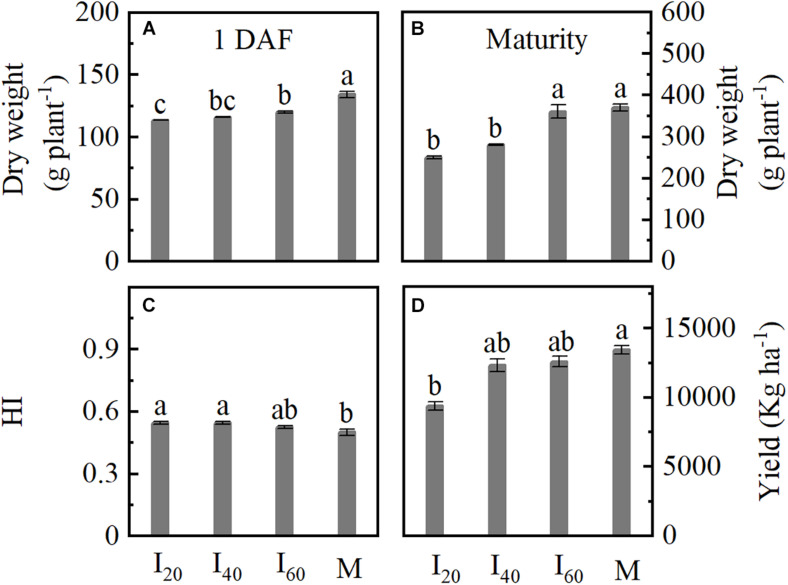
Dry weight, harvest index (HI), and kernel yield of maize in Study 1 (2020). I_20_, I_40_, and I_60_ represent the intercropping maize planted in the narrow–wide row spacing patterns, narrow and wide row spacing of 20 + 200, 40 + 180, and 60 + 160 cm, respectively. M: monoculture maize with a row spacing of 70 cm. **(A,B)** Shoot dry weight at 1 DAF and maturity; **(C)** HI: harvest index; **(D)** kernel yield at maturity. Data with the different letters are significantly different (*p* < 0.05).

We determined the dry weight at 1 DAF and maturity. The dry weight significantly decreased in I_20_ and I_40_ compared to M (Figures 5A,B). I_20_ showed a reduction by 18% for kernel yield compared to M; no significant difference was found between I_40_, I_60_, and M (Figure 5D). The HI in I_20_ and I_40_ were significantly higher than in M (Figure 5C). Hence, the dry weight and yield in smaller narrow-row intercropping (I_20_) were lower than in monoculture maize, but HI increased in intercropping.

### IAA Concentration and Key Biosynthesis Pathway Genes Expression

**FIGURE 6:**
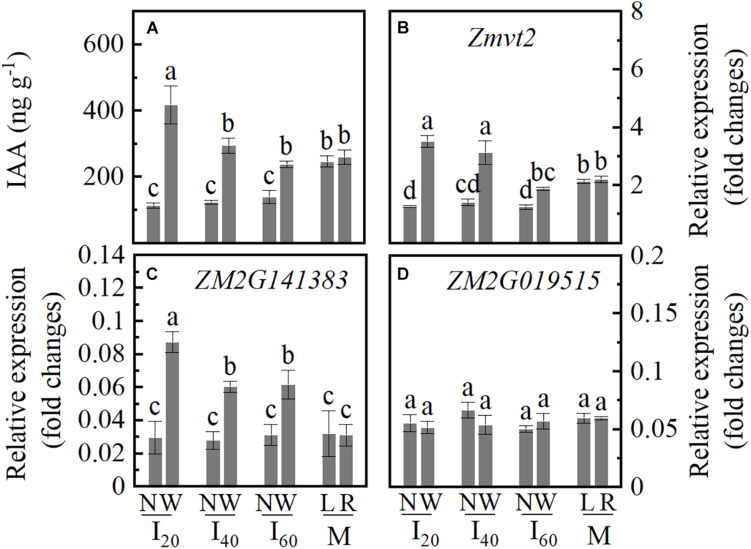
Indole-3-acetic acid (IAA) concentration, relative expression levels of the key genes of IAA biosynthesis in Study 1 (2020). I_20_, I_40_, and I_60_ represent the intercropping maize planted in narrow–wide row spacing patterns, narrow and wide row spacing of 20 + 200, 40 + 180, and 60 + 160 cm, respectively. M: monoculture maize with a row spacing of 70 cm. ML: the left side of monoculture plant; MR: the right side of monoculture plant. N: stem nodes (the first node of aboveground) of narrow row at stage V14; W: stem nodes (the first node of aboveground) of wide row at stage V14. Data with the different letters are significantly different (*p* < 0.05). **(A)** Indole-3-acetic acid (IAA) concentration. **(B–D)** The relative expression levels of Zmvt2, ZM2G141383, and ZM2G01951, respectively.

Indole-3-acetic acid acts as an important regulator in root development; thus, we determined the IAA concentration in the stem nodes in a narrow row (N) and wide row (W). The IAA concentration differences between I_20_N, I_40_N, and I_60_N were not significant; I_20_W was higher than I_40_W, I_60_W, ML, and MR (Figure 6A). *Zmvt2* and *ZM2G141383* genes showed an increased expression level in I_20_W and I_40_W, compared to I_60_W, ML, and MR. The *Zmvt2* expression level decreased in I_20_N, I_40_N, and I_60_N than in ML and MR (Figures 6B,C). However, the expression of the *ZM2G019515* gene did not differ significantly (Figure 6D). Accordingly, in an individual plant, the IAA distribution of narrow and wide rows was not uniform. The IAA concentration in the wide row increased with increasing plant inclination, whiles the IAA accumulation in the narrow row decreased.

### Plant Inclination, BURR, and Brace Root Number in Study 2

**FIGURE 7:**
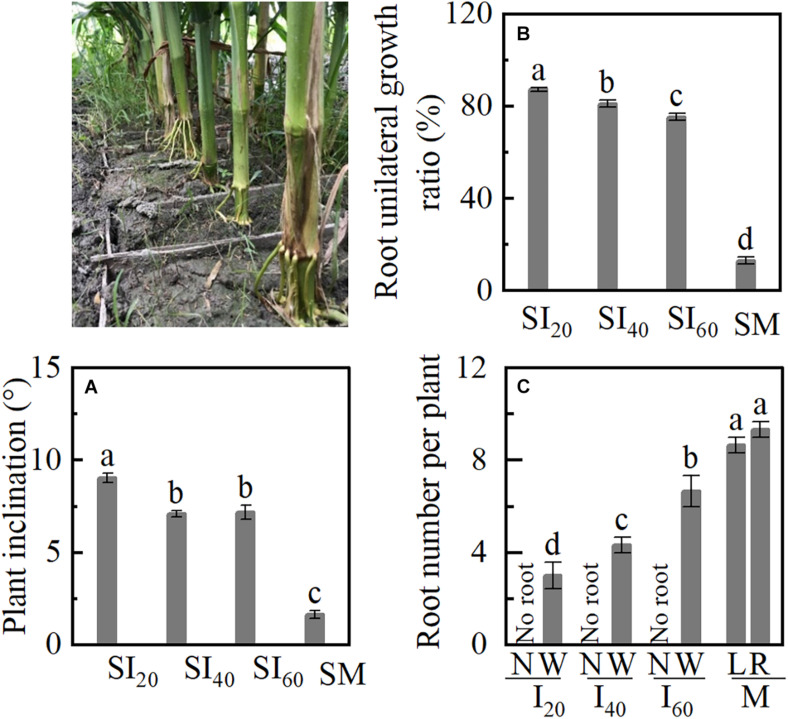
Plant inclination and brace unilateral root growth ratio of a split-root experiment in Study 2. SI_20_: the narrow and wide row spacing of 20 + 200 cm of intercropping maize; SI_40_: the narrow and wide row spacing of 40 + 180 cm; SI_60_: the narrow and wide row spacing of 60 + 160 cm; SM: monoculture maize. **(A)** Plant inclination of maize; **(B)** the brace unilateral root growth ratio. **(C)** The number of brace roots in narrow row and wide row with intercropping and left and right sides of monoculture. Data with the different letters are significantly different (*p* < 0.05).

Plant inclination increased with decreasing narrow-row spacing in a split-root experiment (Figure 7A). SI_20_ inclined more than 9°, and SM inclined 1.6° only. BURR of SI_20_, SI_40_, and SI_60_ were significantly higher than SM, and BURR increased with narrow-row spacing decreasing (Figure 7B). The narrow row (N) had no brace root in SI_20_, SI_40_, and SI_60_, brace root number of wide (W) row decreased with narrow-row spacing decreasing, SI_20_W, SI_40_W, and SI_60_W, were lower than ML and MR. The brace root numbers showed no significant differences between left and right in M (Figure 7C).

### BURR and Brace Root Number in Study 3

**FIGURE 8:**
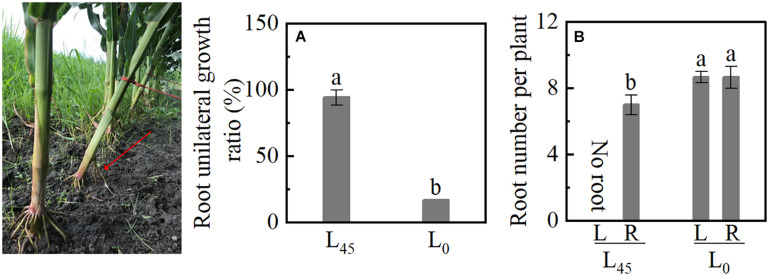
Unilateral root growth ratio of plant inclination experiment in Study 3. L_45_: pull the plant with a rope and tilted it 45°. L_0_: plant erect of maize (L_0_). **(A)** The brace unilateral root growth ratio. **(B)** The number of brace roots on the left and right sides. Data with the different letters are significantly different (*p* < 0.05).

When the plants tilted at 45° (L_45_) was pulled out, the BURR achieved 94%, and the plant’s upright (L_0_) was just 17% (Figure 8A). Although the L_45_ left (L) had no brace root, the number of brace root in L_45_ right (R) was lower than L_0_ left and right; no significant differences were observed between L_0_L and L_0_R (Figure 8B). These results indicated that plant inclination (gravity) leads to brace unilateral root growth and decreasing brace root.

### Correlation Analyses

**FIGURE 9:**
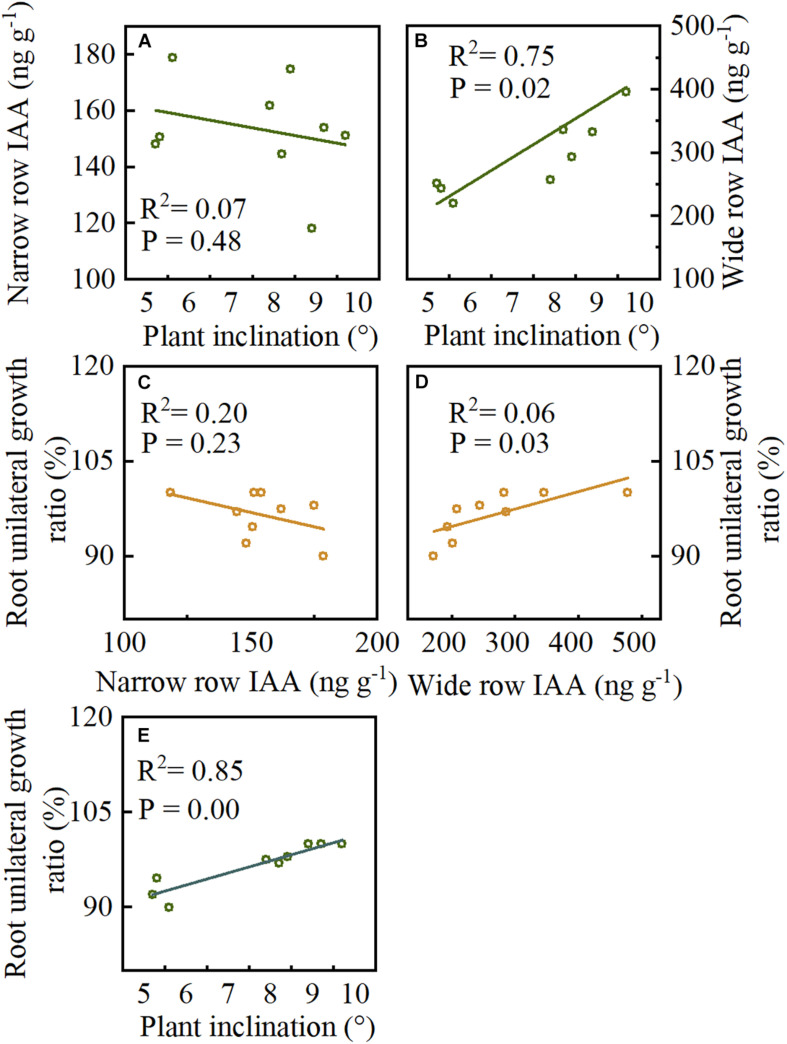
The correlations between plant inclination, IAA, and unilateral root growth ratio were analyzed in Study 1 (2020). **(A,B)** The correlations between narrow row IAA, wide row IAA and plant inclination. **(C,D)** The correlations between narrow row IAA, wide row IAA and root unilateral growth ratio. **(E)** The correlations between plant inclination and root unilateral growth ratio. *p* < 0.05 by Pearson’s correlations. Data forms treatments of I_20_, I_40_, and I_60_, *n* = 9.

Pearson’s correlation was used to determine the correlation between plant inclination, IAA, and unilateral root growth ratio. Plant inclination and narrow-row IAA had no significant correlation, while plant inclination was significantly associated (*p* = 0.02) with wide-row IAA (Figures 9A,B). Moreover, narrow-row IAA showed no significant relationship with unilateral root growth ratio. The wide-row IAA showed a significant positive association (Figures 9C,D, *p* = 0.03). There was a significant positive correlation between root BURR and plant inclination (Figure 9E, *p* = 0.00).

## Discussion

### Gravity Regulated Brace Unilateral Root Growth

Gravity stimulated brace unilateral root growth by increased IAA concentration. It has long been known that water, nutrient, gravity, and light were strongly associated with root growth direction (Morita, 2010; van Gelderen et al., 2018). In the tilted pine seedling, stem reorientation in response to inclination involved hormone regulation such as auxin and jasmonic acid differential distribution on the opposite side of tilted stems (Ramos et al., 2012, 2016; Salazar et al., 2019). However, it is complex research to determine which environmental factors drive these associations in different planting patterns. Fortunately, several studies have revealed that water, spatial heterogeneous fertilizer distribution, and interspecific interaction readjusted root growth direction in intercropping systems (Rahman et al., 2017; Yong et al., 2018; Chen et al., 2019). However, gravity also stimulates root morphology in the maize–soybean intercropping system (MSR). Here, the plant inclination of maize in intercropping was higher than in monoculture, and it increased when narrow-row spacing decreased (Figures 1A, 2). Coincidentally, the BURR increased as narrow-row spacing decreased (Figure 3E). However, these data were inadequate to reveal the causal relationship between BURR and plant inclination. Further root barrier experiment showed that plant inclination and BURR was increased when narrow-row spacing decreased, which was significant in intercropping than in monoculture (Figures 7A,B). Thus, root barrier experiments excluded the water, nutrient, non-uniform distribution impact on unilateral root growth. This failure demonstrates the causal role between plant inclination and unilateral root growth, although BURR was positively associated with plant inclination (Figure 9E). To determine their association, an important plant inclination experiment was designed. The results suggested that almost all the brace unilateral root growth tilted at 45° after pulling the plants using a rope (Figure 8A). Therefore, gravity (plant inclination) is another essential factor for maize unilateral root growth in intercropping.

In practice, many experiments showed that IAA redistributed higher concentration on the lower side of the plant root, lower concentration on the upper side of roots when the root senses gravity (Sato et al., 2014; Suzuki et al., 2016). Some studies indicated that a certain amount of the IAA in the root apex generated from the shoot and another work demonstrated that IAA is synthesized in root apices in *Arabidopsis* (Stepanova et al., 2008; Chen et al., 2014; Yang et al., 2014), and it synthesized in maize root tip region *via* YUC pathway (Suzuki et al., 2016). The tryptophan aminotransferase-related gene and YUCCA genes were involved in IAA synthesis in the maize root apex (Suzuki et al., 2016). Here, we observed that the IAA concentration in stem nodes (nodes develop into brace root) in a wide row increased with plant inclination (Figure 6A). BURR was more than 80% in intercropping (Figures 1B, 3E). In contrast, IAA showed no significant differences in narrow rows in all intercropping treatments (Figure 6A). IAA was not significantly different on both sides of the monoculture plants, while BURR was only 14% in the monoculture. The genetic analysis showed that the *Zmvt2* and *ZM2G141383* (YUCCA gene) of IAA synthesis in wide rows increased with plant inclination. No significant differences were expressed between left and right in monoculture nodes (Figures 6B–D). These results suggest that IAA heterogeneous accumulation can cause brace unilateral root growth. Overall, gravity regulates brace unilateral root growth of maize *via* regulating IAA level in MRS.

### Brace Unilateral Root Growth Reduces N Uptake Ability

In this study, the BURR in intercropping treatments increased than in monoculture, but the root number and surface area were lower than in monoculture (Figures 1B,C,E, 3E,F,J, 7B,C). The greater the BURR, the lesser the root volume, number, weight, and surface area in intercropping (Figures 3E–J). Generally, the brace root will extend and penetrate the soil to keep the plant upright (Zhang et al., 2018) and form root hairs and lateral roots (Supplementary Figure 4) to increase water and nutrient uptake (Hochholdinger et al., 2004). At 25 DAF, the brace root percentage reached the maximum ratio of brace/total root weight and was more than 17% in intercropping and reached 24% in monoculture (Figure 3G). In addition, the leaf, cob, and kernel N accumulation were decreased with BURR increasing in intercropping at maturity in I_20_ and I_40_ that was lower than in monoculture, with no significant differences between I_60_ and monoculture (Figures 1G,I,J, 4C,E,F). Noteworthy phenomena, usual nitrate and ammonium are the mobile nutrient that tends to be concentrated in the soil depth layers, but the brace root distribution in the soil superficial seems difficult for N uptake by the brace root. However, the interaction of late N application and moist soil with low-infiltration capacity tends to the topsoil (van der Bom et al., 2020). In this study, fertilizers were applied for the second time at 10 days before flowering (late N application). With rainfall concentrated after flowering, most N centered on 0–20 cm depth in the MSR system (Chen et al., 2019; Zhou et al., 2019c). Thus, the brace root of the topsoil had a significant contribution to the N uptake task. The brace unilateral root growth reduced the N uptake ability (root volume and surface area), which lead to a decrease in N accumulation after flowering. In contrast, compared to the control, plant inclination of sunflower (self-organized crops) increased oil yield with control crops forced to remain upright (Lopez Pereira et al., 2017). However, in this study with monoculture maize as control, the plant naturally remained upright. The N uptake may increase in intercropping maize if plants remain upright in intercropping as control.

Interestingly, IAA concentration was the highest in I_20_W, whereas the root and weight were minimal. Theoretically, the higher the IAA level, the larger the root number and weight (Ivanchenko et al., 2010; Alarcon et al., 2019). However, root morphology is associated with plant hormones and photosynthate supply from the shoot (Zhou et al., 2019b, c). Moreover, the leaf in narrow row growth in shade stress and photosynthesis decreased (Liu et al., 2018; Chen et al., 2020). Thus, future studies should focus on the root morphology plastic response to photosynthate in MSR. It should be mentioned that we only examined the effect of plant inclination (gravity) on the distribution of brace root and the associations between gravity and N uptake after the flowering stage when the first layer brace root of aboveground started to grow, and the plant began to incline at stage V14 (Supplementary Figure 5). The plant inclination in intercropping was different in sunflower under high-density cultivation. Sunflower plants grew toward one side of the interrow space, while neighboring plants grew in the opposite directions at stage V6 (Lopez Pereira et al., 2017). Therefore, the reason behind the tilted stem growth toward the wide row in intercropping maize requires the further investigation.

## Conclusion

**FIGURE 10:**
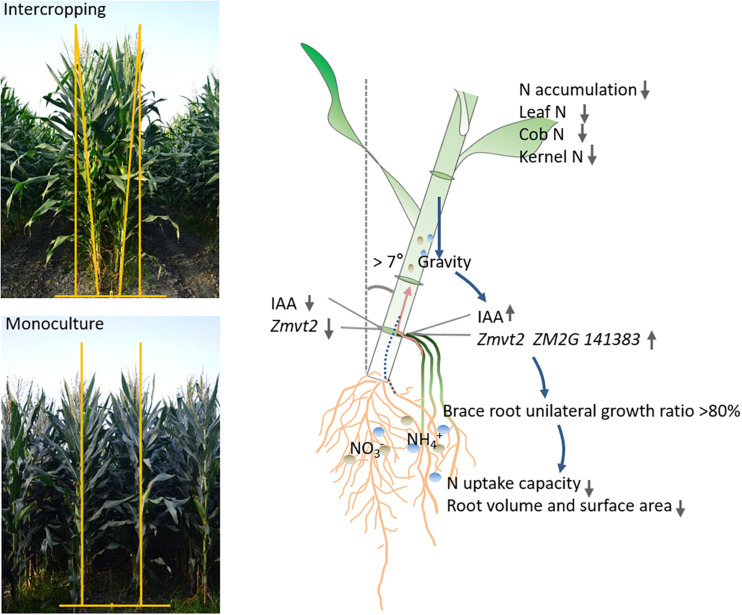
An integrated model of N uptake of intercropped maize response to gravity. The plants tilted toward the wide row, gravity-induced the expression, and upregulation of auxin biosynthesis genes (*Zmvt2* and *ZM2G141383*), IAA accumulation. In more than 80% of plants, the brace root grown in a wide row was absent on the other side. Subsequently, the root volume and surface area were reduced, resulting in decreased N uptake capacity. The leaf, cob, and grain N accumulation were also reduced.

Our results demonstrated that gravity is an important factor in regulating root morphological plasticity. High plant inclination increased the level of IAA in a wide row and BURR, decreased root number, volume, and surface area that decreased the N accumulation in leaf, cob, and kernel. This study provided new insight into root morphology response and N uptake in MSR, which is important in understanding the underlying mechanisms of how gravity regulates distribution of IAA, root growth, nutrient-use efficiency, and yield in an intercropping system (Figure 10). Furthermore, as the plant inclination may be unfavorable for the nutrient uptake and utilization, our results also provide a strategy for row-spacing management in intercropping systems that can help decrease plant inclination and improve root phenotype through row-spacing adjustment.

## Data Availability Statement

The datasets presented in this study can be found in online repositories. The names of the repository/repositories and accession number(s) can be found in the article/Supplementary Material.

## Author Contributions

GC contributed to writing the original draft. BL, HC, and KS contributed to data curation. YH and GB contributed to the methodology. TY and JL contributed to the formal analysis. XS and TP administered the project. YF and PC provided the software. WL and WY supervised the study. JD and FY handled the resources. XW helped in funding acquisition. All authors contributed to the article and approved the submitted version.

## Conflict of Interest

The authors declare that the research was conducted in the absence of any commercial or financial relationships that could be construed as a potential conflict of interest.

## Publisher’s Note

All claims expressed in this article are solely those of the authors and do not necessarily represent those of their affiliated organizations, or those of the publisher, the editors and the reviewers. Any product that may be evaluated in this article, or claim that may be made by its manufacturer, is not guaranteed or endorsed by the publisher.
