# Supplementary material for: Gravity Reduced Nitrogen Uptake via the Regulation of Brace Unilateral Root Growth in Maize Intercropping
Source: Front Plant Sci. 2021 Sep 6;12:724909. doi: 10.3389/fpls.2021.724909 (PMC8450519; doi:10.3389/fpls.2021.724909)
Supplement: Supplementary file 1 [file Data_Sheet_1.docx]

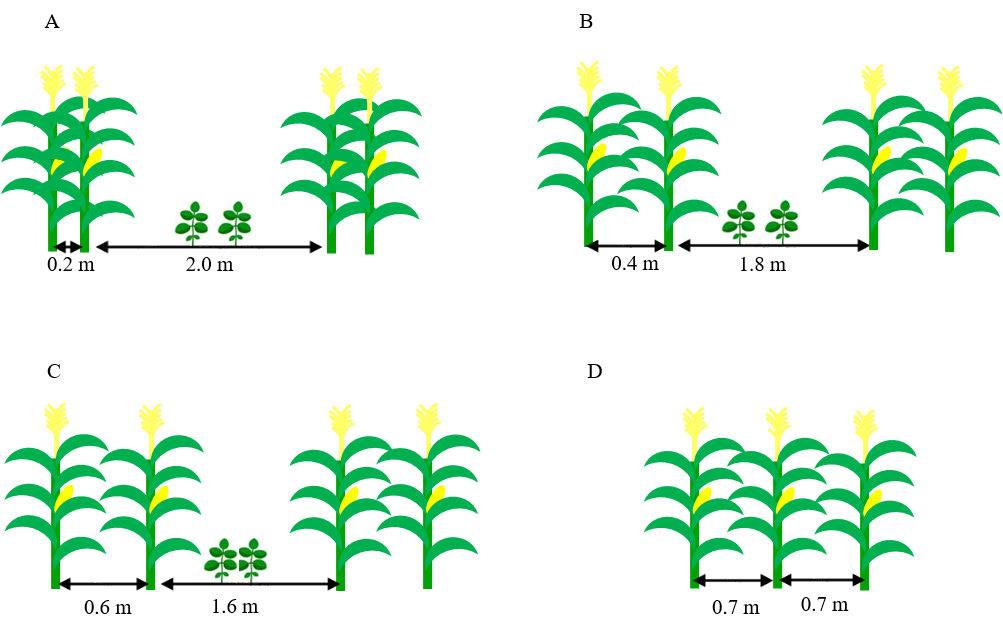


Figure S1. Schematic diagram showing the planting pattern. The narrow and wide row spacing of maize were 20 + 200 cm (I_20_), 40 + 180 cm (I_40_), 60 + 160 cm (I_60_) maize-soybean relay strip intercropping, and monoculture maize (M).


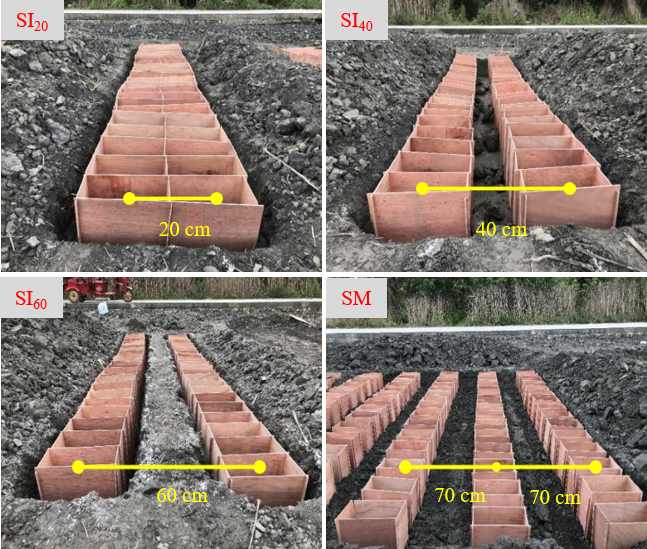


Figure S2. The root of maize barrier picture. Split-root of maize in maize-soybean relay strip intercropping, narrow and wide row spacing were 20 + 200 cm (SI_20_), 40 + 180 cm (SI_40_) and 60 + 160 cm (SI_60_), and monoculture maize (SM).


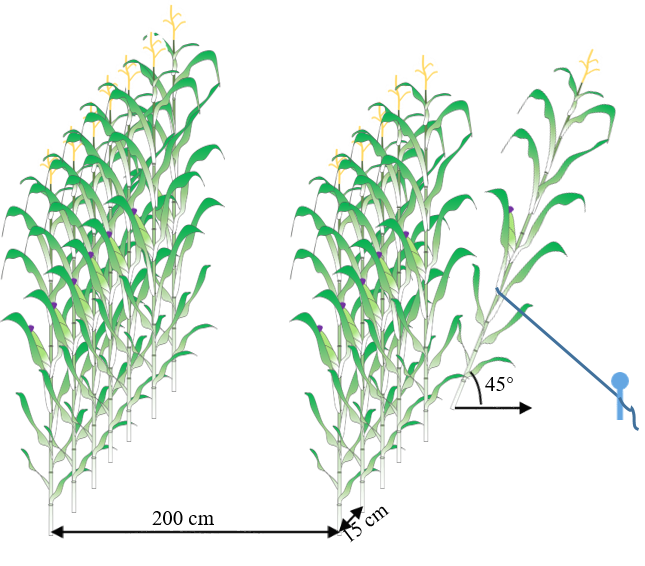


Figure S3. Schematic diagram of plant inclination experiment. The plant was tilted 45° in a vertical direction (L_45_) and erect plant (L_0_).


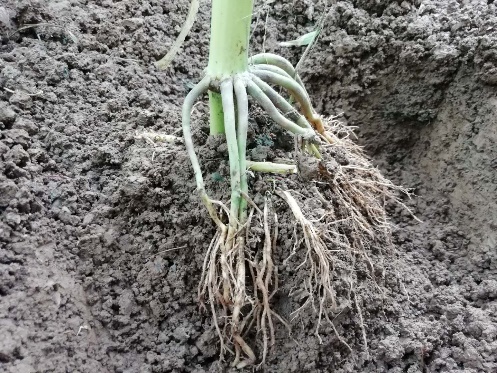


Figure S4. Brace root into the root hair and lateral roots in intercropping maize.


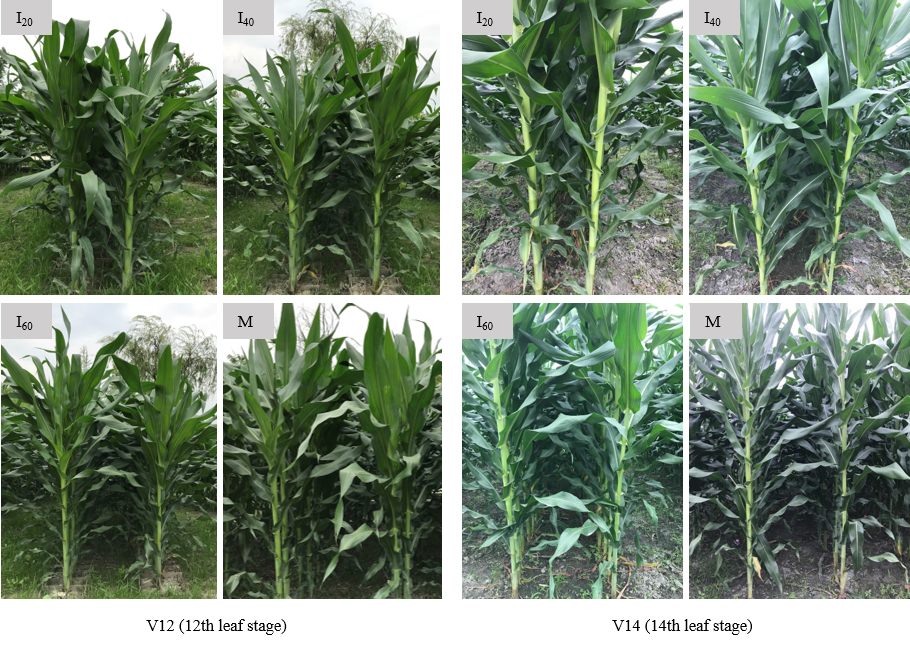


Figure S5. Plant inclination in intercropping and monoculture maize at stages V12 and V14. Plant no inclination both intercropping and monoculture at V12, the plant started to incline at stage V14.

Table S1.

| ID | Forward primer | Reverse primer |
| --- | --- | --- |
| GRMZM2G127308 (vt2) | GCAGCACTAAGCTTAGCTTAGCT | CTCAGCTGAACAGCAGCAGT |
| GRMZM2G141383 | TGGGGCTCCTCCCTACC | GCTCCAGGACGACGAACG |
| GRMZM2G019515 | CTGGAGCGCGACGACTG | GTACTTCTTGGTGAGGTGGAGG |
